# Gray Matter Structural Alterations in Social Anxiety Disorder: A Voxel-Based Meta-Analysis

**DOI:** 10.3389/fpsyt.2018.00449

**Published:** 2018-09-21

**Authors:** Xiuli Wang, Bochao Cheng, Qiang Luo, Lihua Qiu, Song Wang

**Affiliations:** ^1^Department of Clinical Psychology, the Fourth People's Hospital of Chengdu, Chengdu, China; ^2^Department of Radiology, West China Second University Hospital of Sichuan University, Chengdu, China; ^3^Department of Radiology, Huaxi MR Research Center, West China Hospital of Sichuan University, Chengdu, China; ^4^Department of Radiology, the Second People's Hospital of Yibin, Yibin, China

**Keywords:** social anxiety disorder, structural magnetic resonance imaging, gray matter volume, meta-analysis, AES-SDM

## Abstract

The current insight into the neurobiological pathogenesis underlying social anxiety disorder (SAD) is still rather limited. We implemented a meta-analysis to explore the neuroanatomical basis of SAD. We undertook a systematic search of studies comparing gray matter volume (GMV) differences between SAD patients and healthy controls (HC) using a whole-brain voxel-based morphometry (VBM) approach. The anisotropic effect size version of seed-based d mapping (AES-SDM) meta-analysis was conducted to explore the GMV differences of SAD patients compared with HC. We included eleven studies with 470 SAD patients and 522 HC in the current meta-analysis. In the main meta-analysis, relative to HC, SAD patients showed larger GMVs in the left precuneus, right middle occipital gyrus (MOG) and supplementary motor area (SMA), as well as smaller GMV in the left putamen. In the subgroup analyses, compared with controls, adult patients (age ≥ 18 years) with SAD exhibited larger GMVs in the left precuneus, right superior frontal gyrus (SFG), angular gyrus, middle temporal gyrus (MTG), MOG and SMA, as well as a smaller GMV in the left thalamus; SAD patients without comorbid depressive disorder exhibited larger GMVs in the left superior parietal gyrus and precuneus, right inferior temporal gyrus, fusiform gyrus, MTG and superior temporal gyrus (STG), as well as a smaller GMV in the bilateral thalami; and currently drug-free patients with SAD exhibited a smaller GMV in the left thalamus compared with HC while no larger GMVs were found. For SAD patients with different clinical features, our study revealed directionally consistent larger cortical GMVs and smaller subcortical GMVs, including locationally consistent larger precuneus and thalamic deficits in the left brain. Age, comorbid depressive disorder and concomitant medication use of the patients might be potential confounders of SAD at the neuroanatomical level.

## Introduction

Social Anxiety Disorder (SAD), formerly referred to as “social phobia,” is a commonly occurring and highly disabling psychiatric disorder, characterized by an extreme fear of being negatively evaluated in social or performance situations, thus leading to avoidance of social events or enduring them with excessive fear or anxiety (1). SAD usually emerges during early adolescence (2, 3), with a lifetime prevalence of approximately 10–15% (4–6). This disorder shows a rather chronic and unremitting course (7–10), and it is frequently accompanied in later life by comorbid psychopathology such as depression or other anxiety disorders if untreated (11, 12). However, current insight into the development of SAD is still rather limited, hindering the treatment of this disorder. Given that there may be neuroanatomic endophenotypes underlying SAD (13), exploring the neuroanatomical substrates of SAD has the potential to promote the detection and prevention of SAD.

A growing body of neuroimaging literature has investigated the brain structural mechanisms underlying SAD, despite heterogeneous disorder-related findings, with both increased and decreased volume, or cortical thickness, as well as null findings. The first voxel-based morphometry (VBM) study of SAD was reported in 1994 by Potts et al. (14), which failed to find statistically significant volumetric differences in the caudate, putamen and thalamus between 22 SAD patients and 22 controls in an region of interest (ROI) analysis. Another study including 46 patients reported increased cortical thickness in the left insula, right anterior cingulate cortex (ACC) and temporal pole in an ROI analysis, as well as in the right dorsolateral prefrontal and parietal cortex in the whole-brain analysis, while detecting no regions of decreased cortical thickness in the ROI analysis or whole-brain analysis in SAD patients compared with HC (15). Research also pointed toward GM alterations in subcortical regions such as the amygdala and hippocampus in SAD, but these findings often lacked consistency (15–18).

In addition, the inconsistencies of these findings may further be confounded by clinical characteristics associated with brain morphometry, such as age, comorbid depressive disorder and concomitant medications, as well as methodological heterogeneity among studies (19, 20). For example, structural abnormalities in regions implicated in the processing and regulation of fear were reported in pediatric patients with anxiety disorders (21), while one meta-analysis found no significant age effect on GMVs in anxiety disorders (20). The same meta-analysis suggested that comorbid depression might affect the brain anatomical features of anxiety disorders (20). However, only a few studies of SAD have explicitly controlled for depression comorbidity (15, 22, 23). Moreover, treatment studies revealed significant GMV alterations following medication treatment (24, 25), suggesting the influence of psychotropic medication on the GMV alterations in SAD. The reduced GMVs in the prefrontal, parieto-occipital regions and amygdala were observed for SAD patients after effective cognitive behavior therapy (26, 27). Finally, regarding SAD-related alterations in regional GMV, support vector machine (SVM) analyses of gray matter correctly classified SAD participants only when using the whole brain search volume (28).

Thus, the current study performed a coordinate-based meta-analysis of magnetic resonance imaging (MRI) studies using the whole-brain VBM approach to investigate the regional GMV alterations associated with SAD. We also explored the effects of demographic and clinical variables as potential confounders, focusing in particular on the possible impact of age, comorbid depressive disorder and concomitant medication use on the regional GMVs in patients.

## Materials and methods

### Search and inclusion of studies

We conducted a systematic search of the PubMed, Embase, and Web of Science databases for potentially eligible studies that compared GMV differences between SAD patients and healthy controls (HC) and were published in English up to February 2018. A combination of the following key words was used: “structural magnetic resonance imaging OR sMRI OR morphometry OR voxel-based OR voxel-wise OR voxel-based morphometry OR VBM” AND “social anxiety OR social anxiety disorder OR SAD OR social phobia (including public speaking phobia).” Broad search terms were used to minimize the likelihood of missing any relevant studies. We cross-referenced all relevant original research, reviews and meta-analyses, including the reference lists of eligible articles, to identify studies that were potentially missed in the literature searches.

To be considered for inclusion, studies had to meet the following criteria: (1) compared GMV differences between patients with SAD and HC using structural MRI and were published as an original paper in a peer-reviewed journal, (2) included participants from all age groups (due to the relatively small overall number of VBM studies), (3) used a whole-brain voxel-based morphometry (VBM) imaging approach, (4) reported stereotactic coordinates (i.e., Talairach space or Montreal Neurological Institute (MNI) space), (5) enrolled SAD patients–data from samples that included individuals with subclinical social anxiety and autism patients with social anxiety, were not included, and (6) reported *P*-values, so that only differences between groups that met a threshold of *p* < 0.05 (corrected for multiple comparisons) or *p* < 0.001 (uncorrected for multiple comparisons) were deemed significant. For studies based on ROI analyses, we also requested the author(s) provide whole brain results if available. When the same patient group was used in multiple studies, only the study with the largest sample size was selected. If there were several subgroup comparisons, a combined summary result was preferentially included in the meta-analysis. For studies that used longitudinal treatment designs, only baseline pretreatment data were included.

A study was excluded if (1) SAD was investigated solely as a comorbid psychiatric condition or (2) the data were insufficient (e.g., missing neuroanatomical coordinates) even after the author(s) were contacted via email. Finally, we excluded any study that explicitly reported having used (including partially used) data from another published study already included in our meta-analysis.

The process of including literature was as follows. First, two independent reviewers (Xiuli Wang and Bochao Cheng) assessed the titles and abstracts of the search results and retrieved the relevant articles. Second, the full texts of all relevant articles were assessed based on the inclusion and exclusion criteria to determine the included articles. Then, for each study included in the meta-analysis, peak coordinates data of GMV differences found significant at the whole-brain level (no small volume correction, SVC) were independently extracted by two authors (Xiuli Wang and Bochao Cheng) to minimize data extraction errors according to the AES-SDM method (29). Inconsistencies were resolved by a third assessor (Song Wang).

### Voxel-based meta-analysis of regional GMV

A quantitative coordinate-based meta-analysis approach, i.e., the anisotropic effect size version of seed-based d mapping (AES-SDM), allows the results of individual studies to be weighted and controlled for several moderator variables, including demographic, clinical and imaging factors (30). This method has been thoroughly described elsewhere (29, 30) and has been successfully applied to neuropsychiatric populations (31–33).

Using the latest version of AES-SDM (http://www.sdmproject.com/), version 5.141 (30), we analyzed regional GMV alterations in SAD patients compared with HC by a whole-brain VBM imaging approach. This method is briefly summarized here. First, the reported peak coordinates and effect sizes (derived, for example, from *t*-values) of GMV differences were used to recreate, for each study, a map of the effect size of the GMV differences between individuals with SAD and HC. Second, a standard MNI map of the differences in GMV was separately recreated for each study by means of an anisotropic Gaussian kernel with a 20 mm half-width, which assigns higher effect sizes to the voxels more correlated with peaks. Third, the mean map was obtained by voxel-wise calculation of the random-effects mean of the study maps, weighted by sample size, within-study variance and between-study heterogeneity. Division of meta-analytic effect sizes by their standard errors yields *z*-values, but these were not normally distributed; thus, statistical significance was assessed using a permutation test. For all main analyses, it has been shown that *p* < 0.005 (uncorrected) with a cluster-level extent threshold of *k* > 10 optimally balances false positives and negatives (29). For each cluster that was significantly different between patients and controls, Egger's test was used to assess the potential publication bias (34).

Additionally, a jack-knife sensitivity analysis was conducted to assess the robustness of the results by iteratively repeating the mean analysis, excluding one data set at a time, to establish whether the results remained significant (35). In accordance with previous meta-analyses (36, 37), meta-regression analyses were conducted to identify potential demographic and clinical confounders of GMV abnormalities relative to HC, such as the mean age, percentage of male patients, magnetic field strength and image smoothing level within patient groups. We used a more conservative voxel-level threshold of *p* < 0.0005 (uncorrected) and only included findings in regions detected in the main analysis (30, 35). The following variables could not be studied because data were available fewer than nine studies (35, 38): age of onset, duration of illness, and scores on the Liebowitz social anxiety scale (LSAS). Finally, to investigate the potential confounding effect of age, comorbid depressive disorder and concomitant medications, subgroup analyses were performed for studies separately including adult patients, patients without comorbid depressive disorder and currently drug-free patients, followed by jack-knife sensitivity as described above.

### Data extraction

The extracted data included (a) author names, (b) date of publication, (c) subject group numbers, (d) mean age with standard deviation, (e) gender ratio, (f)comorbid depressive disorder, (g) currently concomitant medication, and (h) the coordinates associated with larger or smaller GMVs in the SAD patients compared with the HC.

Peak coordinates were submitted to MRIcron (http://www.nitrc.org/projects/mricron/), which provided templates to visualize the results with MNI coordinates.

## Results

### Included studies and sample characteristics

**Figure 1:**
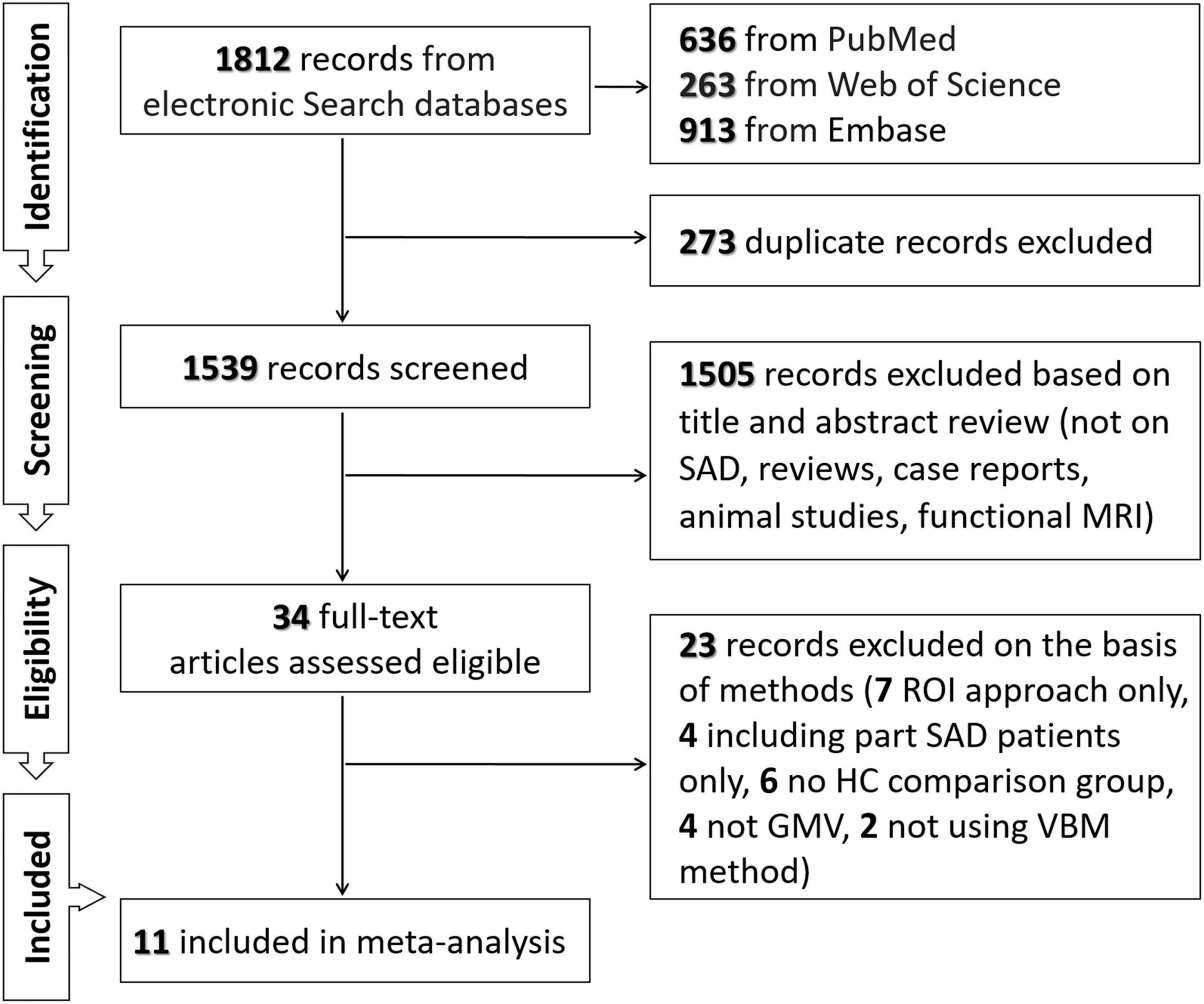
Flowchart of the search strategy and inclusion of studies.

As shown in Figure 1 and Table 1, we identified and included 11 studies (38–48) in the current meta-analysis, comparing the regional GMV differences between 470 SAD patients and 522 healthy subjects at the whole-brain level. In these studies, one mega-analysis (40) collected structural MRI scan data at research centers located in Europe, Africa and North-America. Table 1 presents the characteristics of all included studies. The mean ages of patients (28.67 ± 4.93 years) and healthy subjects (28.18 ± 4.46 years) were not significantly different (*t* = 0.455, *p* = 0.659). The male percentages of SAD patients (243 male patients, 51.70%) and healthy subjects (262 male control subjects, 50.19%) were not significantly different (χ^2^ = 0.226, *p* = 0.340).

**Table 1:** Demographic and clinical characteristics of the SAD participants of the studies included in the meta-analysis (mean ± SD).

**Study**	**Social anxiety disorder patients**								**Healthy subjects**			**Methodology**		
	***N* (male)**	**Age (range)**	**R handedness, n**	**Age of onset**	**Illness duration (y)**	**LSAS**	**Current medications, n**	**Comorbid depression, n**	**N (male)**	**Age (range)**	**R handedness, n**	**Field strength**	**Smoothing (mm)**	**Corrected level**
(39)	20 (13)	23.3 ± 3.7 (NA)	20	NA	47.9 ± 44.1*	52.8 ± 13.7	0	0	30 (21)	26.2 ± 6.6 (NA)	30	3 T	8	FWE *p* < 0.05
(40)	174 (72)	30.6 ± 10.0 (≥18)	172	NA	NA	NA	NA	NA	213 (97)	32.4 ± 10.5 (≥18)	206	3 T	7.5	FWE *p* < 0.05
(41)	48 (24)	33.8 ± 9.3 (NA)	45	NA	NA	72.1 ± 24.0	0	3	29 (13)	23.7 ± 2.0 (NA)	23	NA	8	*p* < 0.05
(42)	67(32)	31 ± 10 (18–70)	62	16 ± 6	15 ± 9	NA	6	NA	64 (33)	32 ± 10 (≥18)	56	3 T	8	*k* > 100, *p* < 0.001 (uncorrected)
(43)	13(5)	36.2 ± 11.8 (20–56)	13	13.0 ± 10.3	23.3 ± 14.4	81.6 ± 14.3	NA	6	18 (18)	33.8 ± 9.6 (>18)	18	3 T	8	FWE *p* < 0.05
(44)	18 (12)	22.7 ± 3.8 (NA)	18	NA	49.2 ± 40.2*	54.4 ± 12.0	0	0	18 (13)	21.9 ± 3.7 (NA)	18	3 T	8	*p* < 0.05
(45)	26 (4)	32.3 ± 9.7 (18–57)	26	15.88 ± 6.0	NA	76.3 ± 18.7	13	NA	26 (8)	32.2 ± 10.5 (≥18)	26	3 T	8	FWE *p* < 0.05
(46)	20 (14)	21.8 ± 3.7 (≥18)	20	NA	50.5 ± 45.8*	52.7 ± 11.7	0	0	19 (13)	21.6 ± 3.7 (≥18)	19	3 T	12	*p* < 0.05
(47)	33 (24)	31.5 (18–50)	NA	NA	NA	NA	0	11	37 (18)	31.4 ± 9.1 (≥18)	NA	1.5 T	8	*p* < 0.05
(48)	27 (12)	27.7 ± 6.7 (18–50)	27	14.52 ± 4.10	13.8 ± 7.0	74.0 ± 26.5	0	0	27 (12)	27.7 ± 5.8 (≥18)	27	1.5 T	8	*p* < 0.05
(49)	24 (15)	24.5 ± 4.0 (18–50)	24	NA	7.6 ± 3.8	57.0 ± 25.5	0	0	41 (26)	27.1 ± 7.2 (≥18)	41	3 T	8	FDR *p* < 0.001

FDR, false discovery rate; FWE, family wise error; LSAS: Liebowitz Social Anxiety Scale; NA, not available; R, right; SAD: patients with social anxiety disorders. *month.

### Regional GMV differences

The exploratory whole-brain VBM analysis revealed significant GMV alterations in SAD patients compared with HC. The SDM value and number of voxels in the case vs. control comparison performed in this meta-analysis are reported in Table 2.

**Table 2:** Clusters showing gray matter differences between SAD and controls in main and subgroup analyses that met our criteria for robustness.

**Brain regions**	**Maximum**					**Cluster**
	**MNI coordinates x, y, z**	**SDM-Z**	***P*-value**	**Voxels**	**BA**	**Breakdown**
**ALL SAD vs. HC**						
**All SAD** > **HC**						
Left precuneus	−2, −54, 48	1.258	0.00124	264		Left precuneus
Right middle occipital gyrus	50, −68, 26	1.199	0.00198	91	39	Right middle occipital gyrus
Right supplementary motor area	12, 14, 58	1.211	0.00177	83	6	Right supplementary motor area
**All SAD**<**HC**						
Left lenticular nucleus, putamen	−24, −2, −8	−1.251	0.00125	607	48	Left lenticular nucleus, putamen
**ADULT SAD vs. HC**						
**Adult SAD** > **HC**						
Right superior frontal gyrus, dorsolateral	12, −18, 66	1.498	0.00008	551	6	Right superior frontal gyrus, dorsolateral
Left precuneus	−2, −56, 52	1.274	0.000748	384	7	Left precuneus
Right angular gyrus	50, −62, 24	1.148	0.001622	174	39	Right angular gyrus
						Right middle occipital gyrus
						Right middle temporal gyrus
Right supplementary motor area	14, 20, 56	1.225	0.00100	134	6.8	Right supplementary motor area
**Adult SAD**<**HC**						
Left lenticular nucleus, putamen	−26, 2, −6	−1.314	0.001209	453	48	Left lenticular nucleus, putamen
Left thalamus	0, −16, 6	−1.234	0.003034	42		Left thalamus
**SAD WITHOUT COMORBID DEPRESSIVE DISORDERS vs. HC**						
**SAD without comorbid depressive disorders** > **HC**						
Left superior parietal gyrus	−20, −48, 68	1.057	0.000217	241	5.7	Left superior parietal gyrus
						Left precuneus
Right inferior temporal gyrus	36, 6, −44	1.056	0.000217	108	20.36	Right inferior temporal gyrus
						Right fusiform gyrus
Right middle temporal gyrus	64, −40, 4	1.057	0.000217	74	21.22	Right middle temporal gyrus
						Right superior temporal gyrus
**SAD without comorbid depressive disorders**<**HC**						
Left thalamus	−2, −20, 2	−1.653	0.000062	471		Bilateral thalamus
**CURRENT DRUG-FREE SAD vs. HC**						
**Current drug-free SAD** > **HC**						
None						
**Current drug-free SAD**<**HC**						
Left thalamus	0, −18, 6	−1.441	0.0007265	316		Left thalamus

*BA, Brodmann's area; HC, healthy control; SAD, social anxiety disorder*.

### GMV differences of all included studies

**Figure 2:**
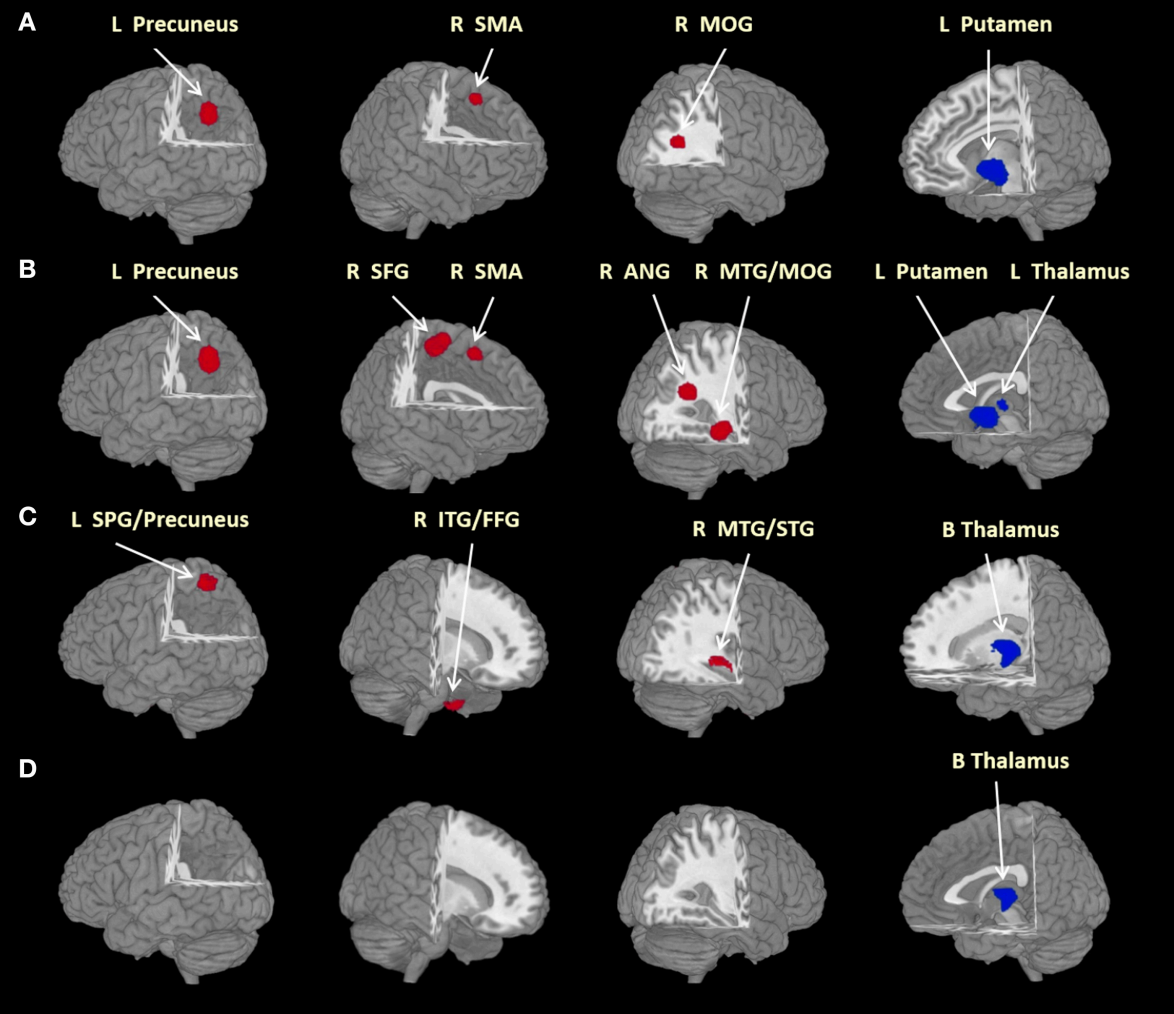
Brain regions differed significantly between groups. Areas of larger (red) and smaller (blue) brain GMVs in patients compared with healthy controls in the meta-analyses. Images are presented in radiological orientation. **(A)** Areas of larger and smaller brain GMVs in all patients with social anxiety disorder compared with healthy controls; **(B)** areas of larger and smaller brain GMVs in adult patients with social anxiety disorder compared with healthy controls; **(C)** areas of larger and smaller brain GMVs in patients without comorbid depressive disorder compared with healthy controls; **(D)** areas of smaller brain GMVs in currently drug-free patients compared with healthy controls. ANG, angular gyrus; B, bilateral; FFG, fusiform gyrus; GMV, gray matter volume; ITG, inferior temporal gyrus; L, left; MOG, middle occipital gyrus; MTG, middle temporal gyrus; R, right; SFG, superior frontal gyrus; SMA, supplementary motor area; SPG, superior parietal gyrus; STG, superior temporal gyrus. Statistical inferences were made with a voxel-level statistical threshold (*p* < 0.005) and a minimum cluster size of more than 10 voxels.

In the main meta-analysis, relative to HC, SAD patients had larger GMVs in the left precuneus, right supplementary motor area (SMA) and middle occipital gyrus (MOG), as well as a smaller GMV in the left putamen (see Table 2 and Figure 2A). Clusters that did not meet the criteria for robustness are shown in Supplementary Table 1.

### Subgroup analyses of sad patients with different clinical features

#### Adult patient comparisons

In eight studies recruiting adult SAD subjects (age > 18 years) with 384 patients and 445 HC, the subgroup analysis revealed that, relative to controls, adult patients with SAD had larger GMVs in the left precuneus, right superior frontal gyrus (SFG), SMA and angular gyrus (extending to the middle temporal gyrus (MTG) and the MOG), as well as a smaller GMV in the left thalamus (Table 2 and Figure 2B). Clusters that did not meet the criteria for robustness are shown in Supplementary Table 2.

#### Patients without comorbid depressive disorder comparison

In five studies recruiting SAD patients without comorbid depressive disorder, including 109 patients and 135 HC, the subgroup analysis revealed that, compared with controls, SAD patients without comorbid depressive disorder had larger GMVs in the left superior parietal gyrus (extending to the precuneus), right inferior temporal gyrus (extending to the fusiform gyrus) and right MTG [extending to the superior temporal gyrus (STG)], as well as smaller GMVs in the bilateral thalami (Table 2 and Figure 2C). Clusters that did not meet the criteria for robustness are shown in Supplementary Table 3.

#### Currently drug-free patient comparisons

In seven studies recruiting SAD patients without current medication use, including 190 patients and 201 HC, the subgroup analysis revealed that, compared with controls, drug-free SAD patients had a smaller GMV in the left thalamus, while no larger GMVs were found (Table 2 and Figure 2D). Clusters that did not meet the criteria for robustness are shown in Supplementary Table 4.

#### Meta-regression analysis

Using a stringent threshold of *p* < 0.0005 to minimize spurious findings, the meta-regression analyses revealed that the alterations of the brain GMV in SAD patients in the main analysis including all studies were not significantly associated with the mean age, percentages of male patients, right-handed patients [available in all studies but one (47)], MRI field strength and image smoothing levels in SAD patients relative to controls.

Insufficient data on age of onset, duration of illness and the symptom dimensions were available to perform meta-regression analysis.

#### Analyses of sensitivity and heterogeneity

As shown in Table 3, a whole-brain jack-knife sensitivity analysis of the meta-analysis indicated that the larger GMV in the left precuneus was preserved throughout the entire dataset. The results of larger GMVs in the right MOG and right SMA, as well as a smaller GMV in the left putamen, remained significant in all but one combination. The analysis of heterogeneity revealed that a number of regions with altered GMVs showed significant statistical heterogeneity among the studies (*p* < 0.005) (Supplementary Tables 5–8).

**Table 3:** Sensitivity analyses of voxel-based morphometry studies of grey matter in patients with SAD in the main meta-analysis.

**Discarded study**	**Gray matter changes**			
	**L precuneus**	**R middle**	**R supplementary**	**L lenticular**
		**occipital**	**motor**	**nucleus**,
		**gyrus**	**area**	**putamen**
(39)	Y	N	Y	Y
(40)	Y	Y	Y	Y
(41)	Y	Y	Y	Y
(42)	Y	Y	N	Y
(43)	Y	Y	Y	Y
(44)	Y	Y	Y	Y
(45)	Y	Y	Y	Y
(46)	Y	Y	Y	Y
(47)	Y	Y	Y	Y
(48)	Y	Y	Y	Y
(49)	Y	Y	Y	N

## Discussion

To the best of our knowledge, this is the first whole-brain voxel-wise meta-analysis exploring GMV alterations in SAD patients using the AES-SDM approach. Overall, our study revealed directionally consistent larger cortical GMVs involving the prefronto-temporo-parieto-occipital cortices and smaller subcortical GMVs in the putamen and thalamus, including locationally consistent larger precuneus and thalamic deficits in the left brain in SAD patients with different clinical characteristics relative to HC. Specifically, relative to controls, we found larger GMVs in locationally different prefronto-temporo-parieto-occipital cortices in all patients with SAD, adult patients with SAD and patients without comorbid depressive disorder, as well as left smaller putamen only in all SAD patients and adult SAD patients. Therefore, our study suggested SAD-related neuroanatomical abnormalities at the whole-brain level and the potential confounding effects of age, comorbid depressive disorder and concomitant medication use by patients. These alterations in brain structures may help explain the dysfunctional processing and regulation of emotion in SAD.

### Overall consistent GMV alterations

Overall, this study revealed directionally consistent larger cortical GMVs mainly involving the prefronto-temporo-parieto-occipital cortices, and subcortical GMV deficits of the putamen and thalamus in SAD patients with different clinical characteristics compared with HC. In line with our findings, one previous SVM study suggested that SAD-related regional GMV alterations were more diffusely distributed over the whole brain (28). In addition, SAD-related abnormalities in brain structure and function may present outside the typical fear circuitry (16, 50), including the amygdala, insula, anterior cingulate and prefrontal cortex (51). The data from structural and functional MRI studies (increased thickness and increased activity) in SAD were rather consistent and pointed in a common direction for some brain regions such as in the prefrontal and temporal cortex (15). Reductions in the prefrontal and parieto-occipital GMV have been associated with treatment response after cognitive behavior therapy for SAD patients (26, 27). Additionally, larger GMVs in SAD patients might reflect a lack of synaptic pruning in an individual. In the mechanisms of structural maturation in the brain, synaptic pruning and myelination may cause developmental reductions of GMV in certain brain regions and improve efficiency in corresponding psychological processes (52–54). An age-related decrease in gene expression involved in synaptic density might be interpreted to suggest decreased cortical GMV in the human brain, especially in the prefrontal cortex (PFC), with advancing age (55). From a network model perspective, both the generation and regulation of emotion were supported by automatic responses in subcortical regions modulated by top-down feedback from the prefrontal regions (56). One study suggested that increasing symptom severity in SAD patients might reflect a growing imbalance between neural mechanisms related to stimulus-driven bottom-up and regulatory top-down processes resulting in dysfunctional regulation strategies (57). Combined with these studies, the altered brain GMVs were likely systematically related to SAD and thus might underlie the alterations in brain functioning consistently reported and replicated in those regions (16, 51, 58, 59). Thus, it is supposed that the increased cortical GMVs in SAD might be the result of continuous efforts to cope with and/or attempts to regulate emotions, i.e., these are compensatory and/or responsive structural alterations for constant anxiety in social or performance situations (60).

Meanwhile, our study found a locationally consistent larger left precuneus (not in the currently drug-free patients) and a thalamic deficit in the SAD patients with different clinical characteristics compared with the HC. A functional imaging study also found that disorder-related scenes, compared with neutral scenes, evoked differential responses in SAD patients in a widespread emotion processing network including limbic structures (e.g., the thalamus) and cortical regions (e.g., the precuneus), which emphasized a central role for the precuneus in disorder-related scene processing (61). Structural and functional abnormalities of the precuneus, a key region of the default mode network (DMN), have been frequently linked to SAD. For example, increased cortical thickness of the precuneus had been reported in SAD individuals compared with HC (15, 22). Abnormal function of the precuneus was also reported in functional neuroimaging studies in patients with SAD (62–64). The putative role of the precuneus has been suggested to promote an individual's tendency to inhibit behaviors and avoid risk related to activation of this region (65, 66). Therefore, one possible explanation of the larger GMV in the left precuneus is a responsive and/or compensatory adaptation to social and performance situations.

Similarly, patients with GAD showed a significantly reduced GMV in the left thalamus compared with HC (67). SAD patients with a higher symptom severity tended to have smaller subcortical volumes, with a trend for lower volume in the left thalamus in SAD patients relative to controls (17). The thalamus is an integral part of the emotion modulation, emotional salience and cognitive/executive networks (68). A previous study reported functional abnormalities of the thalamus during emotion processing in SAD patients (69). Moreover, habituation effects to social stimuli were found in the thalamus of SAD patients (70). Additionally, lesions to this region have been linked to the development of phobias (71). It is assumed that the thalamus belonging to the arousal system may cause anxiety patients to be more easily aroused by emotional stimuli and as a result display exhaustive or decompensated volumetric reduction. Thus, sustained emotional deregulation and failure to inhibit negative affect may lead to progressive atrophy of the thalamus in SAD.

Taken together, the above findings point to a possible anatomical substrate of SAD expressed by GMV abnormalities. The GMV abnormalities might underlie or derive from either a functional disturbance of the cortical regions or a disrupted regulation between the cortical regulating regions and the subcortical targets of regulation in SAD patients. Future studies in SAD populations may target these regions as an a priori focus of investigation to confirm which trait-like GM alterations are typically associated with SAD.

### Specific GMV alterations

When considering the subjects' age, the current research revealed GMV alterations in adult patients with SAD relative to HC, i.e., larger GMVs in the right dorsolateral prefrontal cortex (DLPFC), SMA, angular gyrus, and middle temporal and occipital gyrus as well as a deficit in the left putamen. Our study found similar results in adult patients (age ≥ 18 years) relative to those in the total patients, and no significant age correlation. Thus, it might be suggested that, to a certain extent, the age of the patient group has little effect on the brain GMVs of SAD patients in the current study. Consistent with our findings, some studies have found increased cortical thickness of the right DLPFC, the parietal cortex including the angular gyrus, and the left temporal cortex in adult SAD patients compared with controls (15, 72). Similarly, a meta-analysis demonstrated increased GMVs in the right prefrontal gyrus, precentral gyrus and inferior parietal lobule in adult patients with anxiety disorder (20). Moreover, increased GMV in the prefrontal, temporal and occipital cortices might be related to abnormalities in emotional face processing frequently reported in SAD patients (50). One study also reported age-related negative correlations with GMV in some anatomical brain networks, including the middle frontal gyrus, frontal medial cortex, precuneus, and lateral occipital cortex, in middle-aged to older adults (73). SAD patients showed greater activity than HC in response to disorder-related vs. neutral scenes in brain regions associated with emotion regulation (e.g., DLPFC) and self-referential processing (e.g., the precuneus) (57). Functional connectivity research also suggested an altered interplay between cortical regions (e.g., the PFC and precuneus) in SAD patients (61). Similarly, the increased cortical GMVs in SAD patients might suggest compensatory and/or responsive structural alterations for constant anxiety in social or performance situations.

It was reported that SAD patients showed activations in the right DLPFC, MTG (74) and left inferior occipital gyrus in response to external threat (58). The right DLPFC is known to be more active during emotion suppression (75), which is used more frequently in SAD patients (76). Neuroimaging studies upheld the long-held and popular view that the DLPFC is implicated in emotion regulation circuits (15). Therefore, our study suggested that the right DLPFC might be involved in the neuropathological model in SAD. The SAD participants of the current study presented with brain abnormalities located in the parietal and premotor areas. A study in healthy humans (77) found that the early binding of gaze, gestures and emotions occurs in the premotor cortex, including the supplementary motor cortex and parietal cortex. Avoiding gazing toward emotional stimuli has been repeatedly shown in SAD patients (78). Thus, gaze control might partially explain the larger GMVs in the SMA and angular gyrus in the current study. Exploratory analyses revealed a positive relationship between trait anxiety and brain activation in the SMA during emotional face processing (79). The SMA related primarily to the control of movement but also to fear conditioning (80) and emotion regulation (81). Increased middle/superior temporal gyrus activity was also observed in adult SAD patients during emotional processing compared with HC (79, 82). The right temporal functional activity itself provided the greatest contribution to individual diagnoses of SAD, with an accuracy of 84.5% (83). SAD patients revealed weaker communication of the MTG in the social-affective communication module, proportional to the severity of objective and subjective functional impairment compared to HC (84). The MOG is within the visual recognition network (74) and is involved in the perception of facial emotion (85). SAD is marked by a constant anxiety of facing negative judgement or evaluation in social or performance situations (86). Studies including one meta-analytic review revealed increased resting-state functional connectivity and activities, as well as task-related hyperactivation in the occipital cortex in SAD, which might underlie the enhanced environmental scanning for potentially threatening or feared stimuli in SAD (16, 87, 88). Treatment-related research provided evidence for a link between structural and functional alterations in SAD (45). Interestingly, age-dependent changes in activity were primarily observed in the parieto–temporo–occipital regions in healthy subjects (89). Less regional activity was observed in the prefrontal cortex and supramarginal gyri in the self-face condition, while more regional activity was observed in the prefrontal cortex and angular gyri in the attractive others' face condition in SAD patients than in controls (90). It was suggested that abnormal engagement of the fronto-parietal attentional network during processing face stimuli might be linked to distorted self-recognition in SAD. Thus, the current study might suggest neuroanatomical components of a dysfunctional social-information processing system in adult patients with SAD.

With regard to subcortical structures, and in line with our finding, Potts et al. found an age-related reduction in the putamen volume in SAD patients (14). The putamen, as a part of the striatum, is implicated in cognitive control, social learning and reward processing (91). It has been shown that, compared with HC, patients with SAD lack a processing advantage in the putamen for social rewards relative to social punishments (92). Hence, these findings provided further evidence that structural alterations in the putamen might play a role in the pathophysiology underlying the imbalance in approach-avoidance motivation in SAD [see also (93)].

With comorbid depressive disorder as a potential confounding factor, our study revealed that SAD patients without comorbid depressive disorder had larger GMVs in the right superior, middle and inferior temporal gyrus, fusiform gyrus and left superior parietal gyrus including the precuneus, as well as smaller GMVs in the bilateral thalami compared with controls. In line with our finding, one study identified increased cortical thickness in the right parietal cortex in the whole-brain analysis and, temporal region in the ROI analysis in SAD patients without comorbidities compared with HC (15). Similarly, a thicker inferior temporal cortex including the fusiform gyrus was found in 14 SAD patients (11 without comorbid disorders) compared to the HC (72). Compared with healthy subjects, SAD patients without psychiatric comorbidity exhibited increased neural activities in the superior temporal and intraparietal cortices, and the fusiform gyrus during emotional faces processing (94) and increased activation in the MTG during a social evaluative threat task (95). These findings suggested that comorbid depressive disorder was a potential confounder affecting the GMV alterations in SAD patients, stressing again the need for further research to establish the neuropathological model specifically related to pure SAD.

When considering concomitant medication use, our finding of only the left thalamic GMV deficit in currently unmedicated SAD patients compared with controls is directionally consistent with one pilot study that found cortical thinning in currently untreated SAD patients (22). However, one study reported increased bilateral amygdala and left hippocampus volumes in treatment-naive socially anxious participants compared with controls in an ROI analysis (18). Morphometry studies revealed SAD-related GMV reduction in the bilateral superior temporal, the left inferior parietal and cerebellar cortex following cognitive behavioral group therapy (27) or treatment with escitalopram (24), without correlations of anatomical changes with clinical course (15). Considering that only current drug-free status has been taken into consideration in our study, it is difficult to attribute the finding to direct effects of the medication itself or fully exclude pharmacological-specific sequelae unrelated to SAD. Additionally, combined with small-study effects for the phenomenon that smaller studies sometimes show different, often larger, treatment effects than large ones (96), we need to interpret these findings with caution. Thus, the influence of psychotropic medication on GMV alterations in SAD remains contentious, and concomitant medication use may be among the potential confounding factors.

Taken together, these findings suggest that the age, comorbid depressive disorder and concomitant medication use of SAD patients may affect the anatomical features of SAD. In view of the preliminary findings of our research and their possible relationship with functional alterations in brain reactivity (97), it needs to be further investigated whether the structural abnormalities are specific for SAD itself or whether they reflect risk factors for SAD by controlling these potential confounders.

### Null findings in the limbic structures

It was also of note was that our study found no social anxiety-related amygdaloid and hippocampal volumetric variation (null findings), in line with two studies (15, 22), while inconsistent with others that reported amygdaloid and hippocampal volumetric alterations in patients with SAD (18, 98, 99). A recent review also pointed toward GM density alterations in subcortical regions, such as the amygdala and hippocampus, but often lacked consistency in SAD (13). One possible reason for this inconsistency could be the methodological differences, as these studies conducted ROI-based morphometric analyses. It also may be that VBM analyses have insufficient sensitivity to detect variation in small limbic structures (e.g., the amygdala and hippocampus) (100). For instance, a recent mega-analysis study included in our research found no anxiety-related amygdaloid and hippocampal volume variation in SAD patients using the VBM method (40). Therefore, future research is needed to attentively survey the processes of atrophy/hypertrophy of limbic structures and further determine whether there is a specific correlation of these structures with SAD.

Our study has several limitations, some of which are inherent to the meta-analytic procedure, such as the heterogeneity of the SAD samples between the different studies. First, publication bias is nearly unavoidable despite the efforts we have made to embrace as many unpublished VBM studies and null-findings as possible. The fact that we were not able to fully review all the GMV evidence for SAD was a potential limitation of the present work. Second, our research was limited by the inclusion of relatively small studies on the structural abnormalities of SAD, resulting in limited statistical power. Third, the included studies covered subjects with a wide age range, although the current study performed the subgroup analysis between adult SAD patients and controls and the meta-regression analysis of the age of the patient group. Fourth, we could not determine whether these structural alterations were part of the pathogenesis or a consequence of the disorder because of the cross-sectional nature of the included studies. Moreover, we did not specify the SAD subtypes (specific or generalized) of the patients or how many of them had indicators of other anxiety disorders such as GAD and panic disorder, which might act as potentially confounding factors. Finally, it should also be noted that only current medication status had been taken into consideration. We were not able to directly analyse medication effects in a more fine-grained fashion due to insufficient data.

In conclusion, our findings provided evidence for the involvement of cortical-subcortical GMV alterations in the pathophysiology of SAD. Overall this study revealed directionally consistent larger cortical GMVs and subcortical GMV deficits in SAD patients relative to HC, including locationally consistent larger precuneus and thalamic deficits in the left brain. Age, comorbid depressive disorder and concomitant medication use might be among the potential confounders at the neuroanatomical level of SAD patients. Our findings of the altered neuroanatomical structures may help explain the dysfunctional processing and regulation of emotion in SAD. Prospective and longitudinal studies including homogeneous SAD patients, coupled with uniform multimodal neuroimaging techniques, are needed to elucidate the neuropathological mechanisms underlying SAD and to further clarify the trajectories of neurobiological alterations and their associations with clinical features and specific medication exposure over time.

## Author contributions

XW and SW contributed to the conception of the study. XW, BC, and SW contributed significantly to the analysis and manuscript preparation; XW, BC, LQ, and QL performed the data analyses and wrote the manuscript.

### Conflict of interest statement

The authors declare that the research was conducted in the absence of any commercial or financial relationships that could be construed as a potential conflict of interest.
