# Supplementary material for: Gray Matter Structural Alterations in Social Anxiety Disorder: A Voxel-Based Meta-Analysis
Source: Front Psychiatry. 2018 Sep 21;9:449. doi: 10.3389/fpsyt.2018.00449 (PMC6160565; doi:10.3389/fpsyt.2018.00449)
Supplement: Supplementary file 2 [file Table_2.doc]

**Supplementary Table 2 Clusters showing differences between adult social anxiety disorder and controls did not meet our criteria for robustness**

| **Regions** | **Brodmann areas** | **Peak MNI coordinate x,y,z** | | | **Z** | ***p*** | **Voxels size** |
| --- | --- | --- | --- | --- | --- | --- | --- |
| (undefined) | 48 | 32 | 2 | -16 | -1.768 | <0.001 | 256 |
| (undefined) |  | 0 | -14 | 4 | -1.161 | <0.01 | 70 |
|  |  | 2 | -16 | 0 | -1.152 | <0.01 |  |
