# Supplementary material for: Gray Matter Structural Alterations in Social Anxiety Disorder: A Voxel-Based Meta-Analysis
Source: Front Psychiatry. 2018 Sep 21;9:449. doi: 10.3389/fpsyt.2018.00449 (PMC6160565; doi:10.3389/fpsyt.2018.00449)
Supplement: Supplementary file 3 [file Table_3.doc]

**Supplementary Table 3** Clusters showing differences between social anxiety disorder without comorbid depression and controls did not meet our criteria for robustness

| **Regions** | **Brodmann areas** | **Peak MNI coordinate x,y,z** | | | **Z** | ***p*** | **Voxels size** |
| --- | --- | --- | --- | --- | --- | --- | --- |
| Left superior parietal gyrus | 5 | -20 | -48 | 68 | 1.057 | <0.001 | 87 |
|  |  | -20 | -52 | 62 | 1.054 | <0.001 |  |
|  |  | -24 | -56 | 66 | 1.051 | <0.001 |  |
| Left superior parietal gyrus | 7 | -24 | -52 | 72 | 1.050 | <0.001 | 22 |
| Right inferior temporal gyrus | 20 | 36 | 0 | -44 | 1.045 | <0.01 | 18 |
|  |  | 26 | -4 | -46 | 1.056 | <0.001 |  |
|  |  | 28 | 10 | -44 | 1.056 | <0.001 |  |
| (undefined) | 34 | 20 | -4 | -8 | -1.661 | <0.001 | 23 |
| Right olfactory cortex | 48 | 28 | 8 | -14 | -1.653 | <0.001 | 15 |
| Right lenticular nucleus, putamen | 48 | 30 | 8 | 12 | -1.143 | <0.01 | 325 |
| (undefined) |  | -28 | -10 | -18 | -1.619 | <0.001 | 114 |
|  |  | -22 | -16 | -10 | -1.614 | <0.001 |  |
|  |  | -16 | -12 | -12 | -1.613 | <0.001 |  |
|  |  | -22 | -10 | -20 | -1.610 | <0.001 |  |
|  |  | -18 | -10 | -18 | -1.609 | <0.001 |  |
