# Supplementary material for: Gray Matter Structural Alterations in Social Anxiety Disorder: A Voxel-Based Meta-Analysis
Source: Front Psychiatry. 2018 Sep 21;9:449. doi: 10.3389/fpsyt.2018.00449 (PMC6160565; doi:10.3389/fpsyt.2018.00449)
Supplement: Supplementary file 4 [file Table_4.doc]

**Supplementary Table 4** Clusters showing differences between social anxiety disorder without current medication and controls did not meet our criteria for robustness

| **Regions** | **Brodmann areas** | **Peak MNI coordinate x,y,z** | | | **Z** | ***p*** | **Voxels size** |
| --- | --- | --- | --- | --- | --- | --- | --- |
| Left cuneus cortex | 18 | -2 | -88 | 16 | 1.158 | <0.01 | 58 |
|  |  | 0 | -86 | 20 | 1.158 | <0.01 |  |
|  |  | -4 | -76 | 22 | 1.157 | <0.01 |  |
|  |  | 0 | -76 | 24 | 1.154 | <0.01 |  |
|  |  | 4 | -86 | 18 | 1.14 | <0.01 |  |
| Right cuneus cortex | 18 | 6 | -78 | 22 | 1.152 | <0.01 | 24 |
|  |  | 6 | -74 | 20 | 1.147 | <0.01 |  |
|  |  | 4 | -82 | 22 | 1.146 | <0.01 |  |
| (undefined) |  | 8 | -74 | 10 | 1.157 | <0.01 | 32 |
|  |  | -8 | -80 | 10 | 1.156 | <0.01 |  |
|  |  | -8 | -76 | 10 | 1.155 | <0.01 |  |
|  |  | 8 | -74 | 16 | 1.144 | <0.01 |  |
|  |  | 10 | -78 | 12 | 1.123 | <0.01 |  |
| Left calcarine fissure / surrounding cortex | 17 | -4 | -84 | 4 | 1.153 | <0.01 | 39 |
|  |  | -4 | -70 | 14 | 1.153 | <0.01 |  |
|  |  | 2 | -86 | 6 | 1.146 | <0.01 |  |
| Left calcarine fissure / surrounding cortex | 18 | -6 | -88 | -10 | 1.157 | <0.01 | 34 |
| Right calcarine fissure / surrounding cortex | 17 | 4 | -70 | 10 | 1.146 | <0.01 | 30 |
| Right calcarine fissure / surrounding cortex | 18 | 4 | -70 | 18 | 1.152 | <0.01 | 14 |
| Left lingual gyrus | 17 | -2 | -76 | 2 | 1.153 | <0.01 | 34 |
|  |  | 2 | -82 | 2 | 1.148 | <0.01 |  |
|  |  | 0 | -70 | 6 | 1.144 | <0.01 |  |
| Right lingual gyrus | 18 | 12 | -74 | -10 | 1.146 | <0.01 | 61 |
|  |  | 20 | -76 | -12 | 1.145 | <0.01 |  |
| Left cerebellum, crus I | 18 | -14 | -90 | -22 | 1.158 | <0.01 | 53 |
| Left cerebellum, crus I | 37 | -46 | -56 | -28 | 1.157 | <0.01 | 71 |
| Left inferior temporal gyrus | 37 | -48 | -54 | -24 | 1.156 | <0.01 | 45 |
|  |  | -54 | -58 | -22 | 1.153 | <0.01 |  |
|  |  | -54 | -62 | -22 | 1.151 | <0.01 |  |
| Left fusiform gyrus | 37 | -48 | -60 | -20 | 1.155 | <0.01 | 15 |
| Left hippocampus |  | -20 | -12 | -12 | -1.443 | <0.001 | 44 |
|  |  | -22 | -10 | -16 | -1.423 | <0.001 |  |
| Left lenticular nucleus, putamen | 48 | -26 | 2 | -6 | -1.144 | <0.01 | 69 |
| (undefined) |  | 2 | -20 | 2 | -1.439 | <0.001 | 27 |
| (undefined) |  | -4 | 36 | -30 | -1.159 | <0.01 |  |
|  |  | -2 | 36 | -30 | -1.159 | <0.01 |  |
| (undefined) | 11 | -2 | 36 | -28 | -1.159 | <0.01 | 11 |
|  |  | -4 | 36 | -28 | -1.159 | <0.01 |  |
