# Supplementary material for: Gray Matter Structural Alterations in Social Anxiety Disorder: A Voxel-Based Meta-Analysis
Source: Front Psychiatry. 2018 Sep 21;9:449. doi: 10.3389/fpsyt.2018.00449 (PMC6160565; doi:10.3389/fpsyt.2018.00449)
Supplement: Supplementary file 8 [file Table_8.doc]

**Supplementary Table 8** Clusters showing significant between study heterogeneity in social anxiety disorder without current medication

| **Regions** | **Brodmann areas** | **Peak MNI coordinate x,y,z** | | | **Z** | ***p*** | **Voxels size** |
| --- | --- | --- | --- | --- | --- | --- | --- |
| Left lenticular nucleus, putamen | 48 | -26 | 2 | -6 | 5.743 | <0.001 | 174 |
| (undefined) |  | -4 | 36 | -30 | 5.907 | <0.001 | 80 |
| Right lenticular nucleus, putamen | 48 | 32 | -6 | -4 | 5.167 | <0.001 | 94 |
